# Light control of G protein signaling pathways by a novel photopigment

**DOI:** 10.1371/journal.pone.0205015

**Published:** 2018-10-01

**Authors:** Tomás Osorno, Oscar Arenas, Nelson J. Ramírez-Suarez, Fabio A. Echeverry, María del Pilar Gomez, Enrico Nasi

**Affiliations:** 1 Departamento de Biología, Universidad Nacional de Colombia, Bogotá, Colombia; 2 Marine Biological Laboratory, Woods Hole, Massachusetts, United States of America; 3 Instituto de Genética, Universidad Nacional de Colombia, Bogotá, Colombia; Doheny Eye Institute/UCLA, UNITED STATES

## Abstract

Channelopsins and photo-regulated ion channels make it possible to use light to control electrical activity of cells. This powerful approach has lead to a veritable explosion of applications, though it is limited to changing membrane voltage of the target cells. An enormous potential could be tapped if similar opto-genetic techniques could be extended to the control of chemical signaling pathways. Photopigments from invertebrate photoreceptors are an obvious choice—as they do not bleach upon illumination -however, their functional expression has been problematic. We exploited an unusual opsin, pScop2, recently identified in ciliary photoreceptors of scallop. Phylogenetically, it is closer to vertebrate opsins, and offers the advantage of being a bi-stable photopigment. We inserted its coding sequence and a fluorescent protein reporter into plasmid vectors and demonstrated heterologous expression in various mammalian cell lines. HEK 293 cells were selected as a heterologous system for functional analysis, because wild type cells displayed the largest currents in response to the G-protein activator, GTP-γ-S. A line of HEK cells stably transfected with pScop2 was generated; after reconstitution of the photopigment with retinal, light responses were obtained in some cells, albeit of modest amplitude. In native photoreceptors pScop2 couples to G_o_; HEK cells express poorly this G-protein, but have a prominent Gq/PLC pathway linked to internal Ca mobilization. To enhance pScop2 competence to tap into this pathway, we swapped its third intracellular loop—important to confer specificity of interaction between 7TMDRs and G-proteins—with that of a G_q_-linked opsin which we cloned from microvillar photoreceptors present in the same retina. The chimeric construct was evaluated by a Ca fluorescence assay, and was shown to mediate a robust mobilization of internal calcium in response to illumination. The results project pScop2 as a potentially powerful optogenetic tool to control signaling pathways.

## Introduction

Controlling cellular activity by exogenous stimulation can help unravel the functioning of cell ensembles and the neural control of behavior, and holds great promise for therapeutic intervention. Since the pioneer work of Rasmussen and Penfield [1], the dominant approach has been electrical stimulation, but its limitations are severe: surface electrodes in intact tissue lack specificity, whereas tissue penetration for application of more focal stimuli is necessarily invasive. Moreover, with extracellular electrical stimulation it is virtually impossible to selectively target cells of a defined type within a mixed population. The discovery that the phototropic response in the unicellular alga *Chlamydomonas* is initiated by proteins that operate simultaneously as light-receptors and ion channels opened a new horizon: these proteins, baptized channelopsins, were cloned, and functional heterologous expression was obtained [2, 3]. Targeted channelopsin expression driven by a specific promoter can make a particular cell type selectively susceptible to control by light [4]. The novel technology proved robust, spawning a veritable explosion of applications, ranging from functional mapping of neuronal networks in excised tissue, to behavioral control in intact animals [5]. The range of possible voltage manipulations subsequently expanded to include inhibitory effects, either by using light-driven pumps [6, 7], or re-engineering the ion selectivity of channelopsins [8, 9]. The immense potential of this approach naturally leads to the question of whether optical manipulation of cells can be extended in scope, to exert control over chemical signaling pathways. Among these, G-protein-mediated enzymatic cascades are especially ubiquitous and important for regulating a plethora of cellular functions. Even for controlling the electrical activity of the target cells, G-protein pathways can be enlisted to exert a wide spectrum of modulatory influences on ion channels, altering, for example, open times [10] or inactivation [11]. This general goal could be attained by utilizing an exogenously implanted 7-transmembrane receptor (7TMDR), whose activity could be controlled by light. Ingenious efforts in this direction have surfaced, like using a metabotropic glutamate receptor conjugated to an azobenzene-derived photoactivatable linker to which an agonist molecule has been attached: light-induced conformational transitions of the linker bring the agonist moiety close to or far from its binding site, allowing reversible light control of the receptor and its cognate G-protein pathway [12]. This strategy is powerful, but complex: because neither the linker nor the agonist are proteic, they are introduced after expression of the suitably modified 7TMDR, which typically incorporates engineered cysteines to serve as acceptor of the linker-agonist complex via thiol chemistry. These additional steps reduce the generality and practical applicability of such approach.

A more straightforward alternative would be to use photopigments from visual cells—which signal through G-proteins—but there are hurdles to be overcome. Mammalian rhodopsin has been functionally expressed [13], but, because it bleaches after photoisomerization, repetitive regeneration is required; this limitation also applies to chimeric constructs comprised of portions of vertebrate rhodopsin and of a metabotrobic receptor [14,15]. Thermally stable photopigments—like those of invertebrates—offer a critical advantage in this regard. However, although numerous photopigments from invertebrate eyes have been cloned [16], heterologous expression has been problematic, and so far only the rhodopsin of the Japanese honeybee appears amenable [17]. This prompted the suggestion that such opsins may require a particular complement of additional proteins in the host cell for proper folding and chromophore binding; in support of this notion, *Limulus* rhodopsin transcripts introduced into *Xenopus* oocytes proved ineffective, whereas poly-A mRNA from the eye successfully confers light sensitivity [18]; likewise, it has been possible to express insect rhodopsins using as host another insect photoreceptor cell in which the appropriate machinery exists [19, 20]. To overcome such limitations, co-expression of multiple photoreceptor-specific proteins has been implemented to produce light responses in *Xenopus* oocytes and in cultured neurons [21], an approach that is inevitably cumbersome. In a recent breakthrough enkephalopsin (a.k.a. panopsin) homologs of pufferfish and mosquito could be hererologously expressed [22], and were shown not to suffer photobleaching. *In vitro* assays demonstrated that illumination of such purified photopigments promotes GTP-γ-S binding to the α-subunit of G_i_ and G_o_; moreover, light decreases cAMP levels in transfected HEK cells, indicating that implanted panopsins are competent to activate their cognate G-protein signaling pathways.

Of particular interest is the proposition that a reversible optical switch be generated, capable of turning ‘on’ or ‘off’ a given G-protein cascade. Such goal could be accomplished with the bi-stable photopigments of certain invertebrates, in which the rhodopsin-metarhodopsin conformational change is accompanied by a large shift in the absorption spectrum, so that transitions in either direction can be induced by varying the chromatic content of the light stimulus (reviewed by [23]). There remains the difficulty of heterologously expressing invertebrate photopigments. Because such hurdle has not been encountered with vertebrate opsins [13, 22], the odds could be more favorable for opsins belonging to a lineage closer to those of vertebrates. Such may be the case of the light-sensing molecules of distal photoreceptors of *Pectinidae*. These are ciliary, hyperpolarizing visual cells that utilize a light-transduction cascade that diverges from that of rods and cones [24–25]. The first putative photopigment of this kind was cloned in the giant scallop *Mizuhopecten yessoensis* [26] and dubbed Scop2. A closely related form was recently molecularly identified in the bay scallop (*Pecten irradians*), a model system extensively used for single-cell electrophysiology; an RNAi approach was utilized to directly confirm that it forms the functional photopigment which underlies the light response of the ciliary photoreceptors, and also that it signals through G_o_ [27]. The primary sequence of Scop2 differs profoundly from the rhodopsins of microvillar photoreceptors (R-opsins). Instead, it forms a sister group of vertebrate photopigments [26,28]. This type of visual opsin may therefore hold greater promise as a genetically implantable, reversible switch for controlling G-protein cascades.

## Results

**Fig 1:**
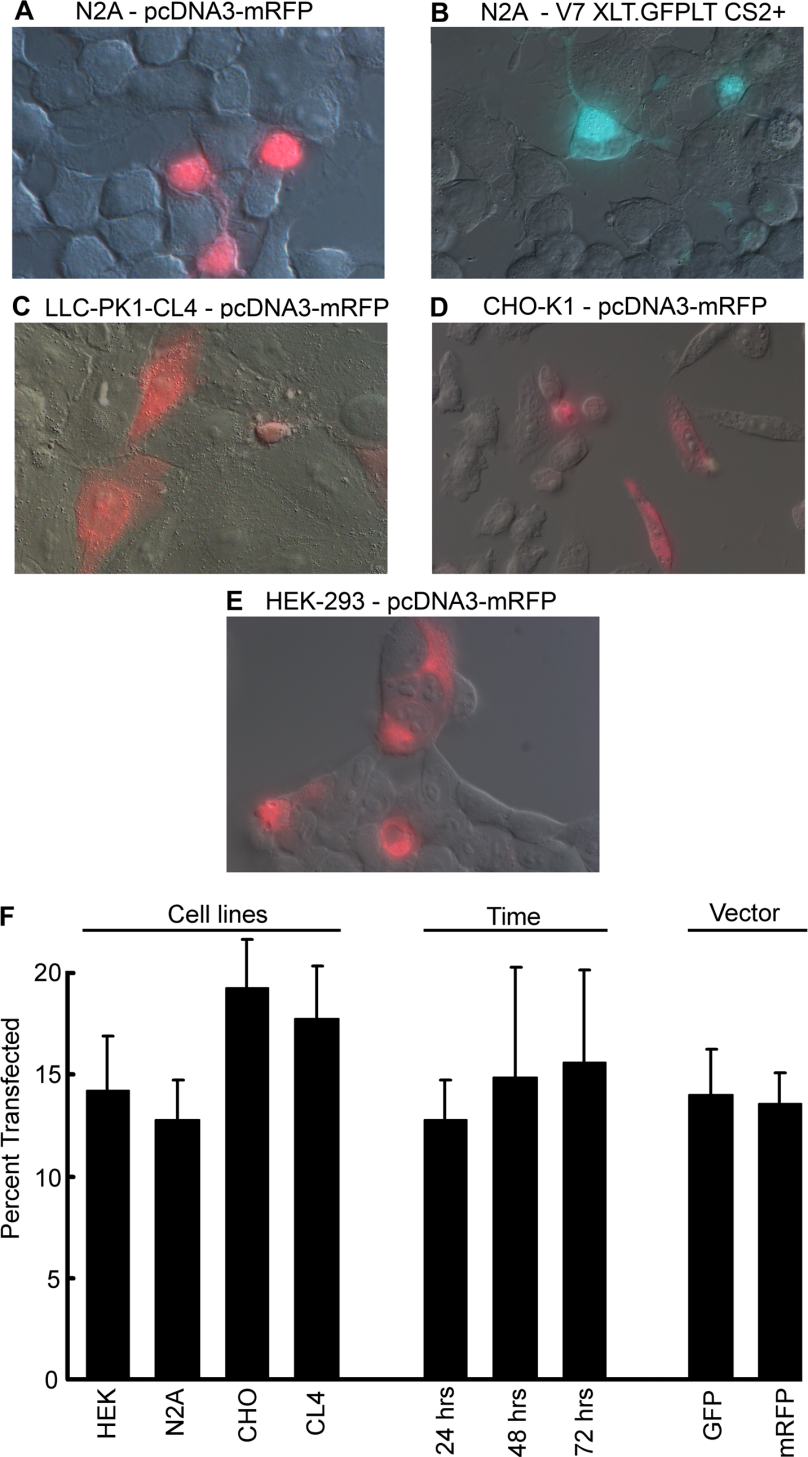
**Expresssion of pScop2 as a fusion construct with either GFP or mRFP as a reporter, in different mammalian cell lines (Panels A-E)**. Cells were examined 24 hours after transfection by lipofectamine. Nomarski and fluorescence images were overlaid. (F) Bar graph representing the efficiency of transfection, as determined from cell counts derived from multiple fields (error bars indicate SEM). The first group of bars compares the different cell lines (left); the middle group shows the percent transfected N2A cells at three time points (24, 48, and 72 hrs.). An overall comparison of the efficiency of the two vectors is displayed in the right portion of the graph.

Although the first member of the class of G_o_-coupled opsins, Scop2, was molecularly identified quite some time ago [26], heterologous expression had not been reported. Having recently cloned an ortholog in the related species, *Pecten irradians* (dubbed pScop2, Genebank Accession number MG674154) and demonstrated that it mediates the light-response in ciliary photoreceptors [27], we assayed heterologous expression by lipofectamine transfection of this new member of the group in several mammalian cell lines (N2A, HEK292, CHO, and LLC-PK1-CL4T), in combination with two expression vectors that encoded pScop2 in a fusion construct with either GFP or mRFP (vectors V7 XLT.GFPLT CS2+ and pcDNA3-mRFP). The purpose of these multiple comparisons was to find conditions that provide reasonable efficiency of expression and localization of the fluorescent construct at least partly in the plasma membrane. With all host/vector combinations a fraction of the cells, examined 24 hrs after transfection, showed distinct fluorescence, whereas all control plates were completely devoid of marker. (Fig 1A–1E) shows superimposed fluorescence and DIC micrographs of representative fields for the different cell types. The average percentage of positive cells is displayed in the leftmost part of the bar-graph of panel F. There were only minor differences across cell types, with a slight trend indicating that in CHO cells the success of transfection was somewhat greater (efficiency hovering around 20%). For N2A cells, the time course of expression was assessed at 3 time points: there was only a marginal increase at 48 hrs (14.9% *vs*. 12.8%), and essentially no sign of further improvement at 72 hrs. (15.6%; middle bars of panel F). Also, there was no systematic difference between the two vectors, as illustrated by panels A and B of (Fig 1) and by the rightmost portion of (Fig 1F) (14% *vs*. 13.6% for the GFP and the mRFP vector, respectively, pooling the various cell types). With regard to the spatial distribution of the fluorescence, it was usually widespread, but slight disparities were observed, with accumulation in the nucleus and at the plasma membrane prevailing in N2A cells (Fig 1A and 1B), and often in HEK293, whereas in the case of CHO and CL4 the fluorescence tended to be more homogeneous (Fig 1, Panels C and D).

### Ascertaining G protein-coupled conductances

**Fig 2:**
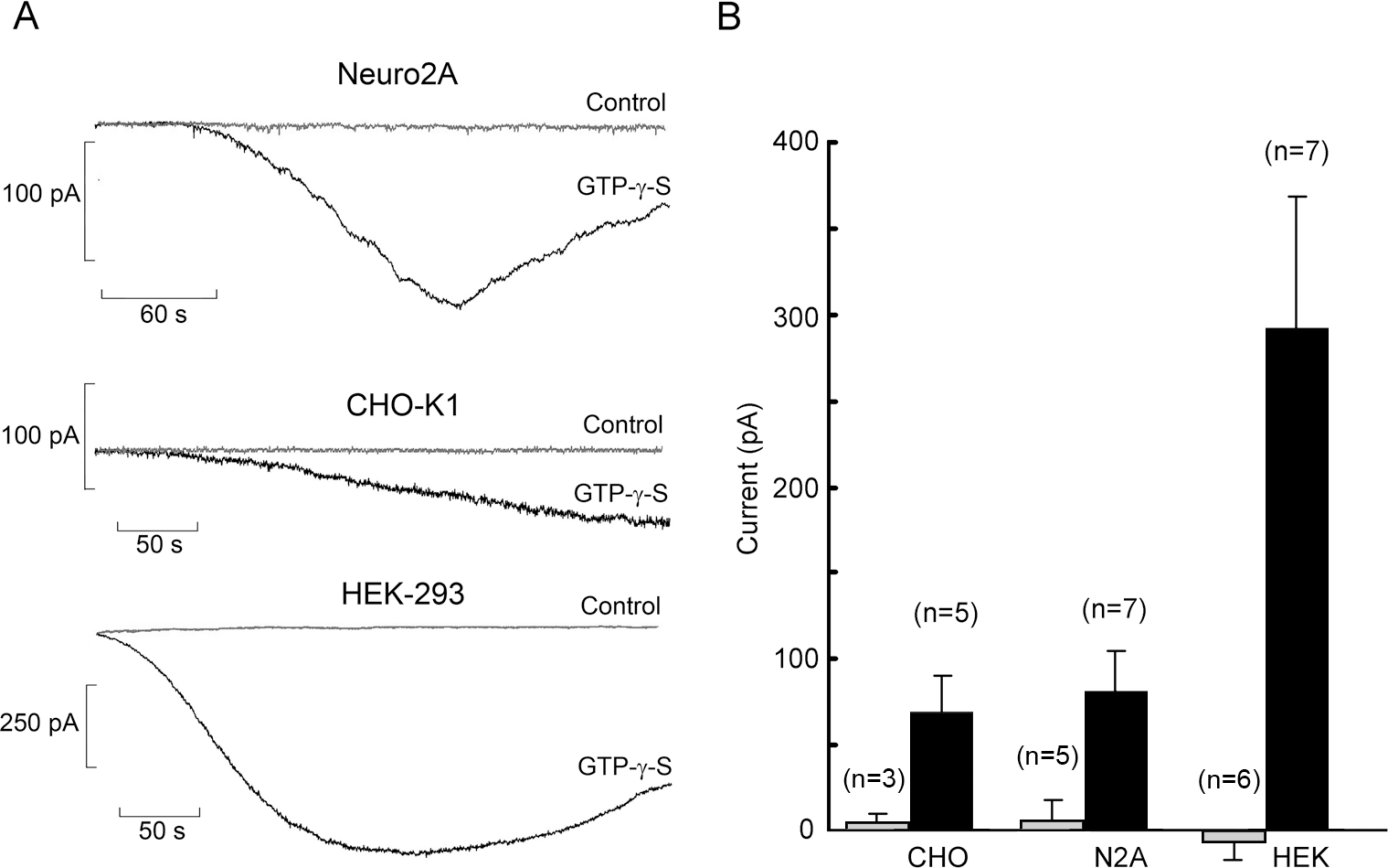
Membrane currents evoked by activation of hetrotrimeric G proteins in wild-type cell lines. (A) The activator GTP-γ-S was applied intracellularly by perfusion via the patch pipette, at a concentration of 100 μM. In all three cell lines tested, GTP-γ-S evoked an inward current with a slow time course; control recordings obtained with GTP (grey traces) remained flat. (B) Bar-graph comparing the average peak amplitude of the GTP-γ-S-elicited current in Neuro2QA, CHO-K1, and HEK 293 cells (black bars; error bars indicate standard deviation); HEK cells consistently produced a larger response. For control cells of each type (grey bars) the change in holding current between the beginning and end of the recording period was measured.

To assess the functionality of the heterologoulsy expressed pScop2 photopigment of *Pecten* ciliary photoreceptors we first turned to an electrophysiological assay. Pilot patch-clamp recordings in wild-type cells lead us to discard the CL4 line: although these cells have been deemed to be particularly favorable for the correct membrane targeting of certain implanted proteins [29], this did not seem the case with pScop2-mRFP (see Fig 1C), and their extremely flat morphology proved too formidable for routine patch-clamp use. An additional requirement is that the host cells be endowed with an endogenous G-protein pathway coupled to a membrane conductance, so that activation of an implanted 7TMD receptor like pScop2 could be monitored electrically. We screened the remaining cell lines using the poorly hydrolyzable GTP analog GTP-γ-S [30, 31], dialyzed intracellularly via the patch pipette (100 μM, replacing GTP). (Fig 2A) shows representative traces recorded in wild-type non-transfected HEK, CHO and N2A cells held at -50 mV: in all cases, shortly after accessing the cell interior, an inward membrane current gradually developed; control recordings without GTP-γ-S were totally flat (grey traces). Significant differences in current size were observed across the three cell types: the bar-graph in Fig 2B summarizes the pooled data, underscoring the much larger GTP-γ-S-evoked current in HEK cells (amplitudes in excess of 600 pA were measured in some cells). A one-way non-parametric statistical comparison by the Kruskal-Wallis test revealed that the differences were significant (p = 0.0122). These results lead us to focus henceforth on this cell line to test the functionality of implanted pScop2.

### Functional expression

To assess possible light effects in transfected HEK cells, the photopigment must be reconstituted. The native chromophore of pScop2 is presently not known, but that of a related opsin of *Branchiostoma*, Amph OP1, has been identified as 11-*cis*-retinal [32]. We used primarily this chromophore to reconstitute the photopigment, but, because of its very limited availability, in several assays we also tried 9-*cis*-retinal, which is commercially available, and has been successfully employed in some systems [33]. In addition, since pScop2 does not bleach with light, *all-trans*-retinal was tested too: because the photo-isomerized chromophore remains attached to the protein moiety, it seemed plausible that exogenously supplied *all-trans*-retinal ought to bind to the free opsin. The reporter of choice for these studies was mRFP, because the 540–570 nm epi-illumination offers two advantages: (i) the absorbance by pScop2 in that spectral band is nearly ten-fold lower, compared to light that optimally excites GFP; therefore, identification of transfected cells by fluorescence may have fewer detrimental effects. (ii) Such illumination is of longer wavelength than the isosbestic point of the photopigment (530 nm) and would therefore tend to photo-restore rhodopsin from metarhodopsin [34].

**Fig 3:**
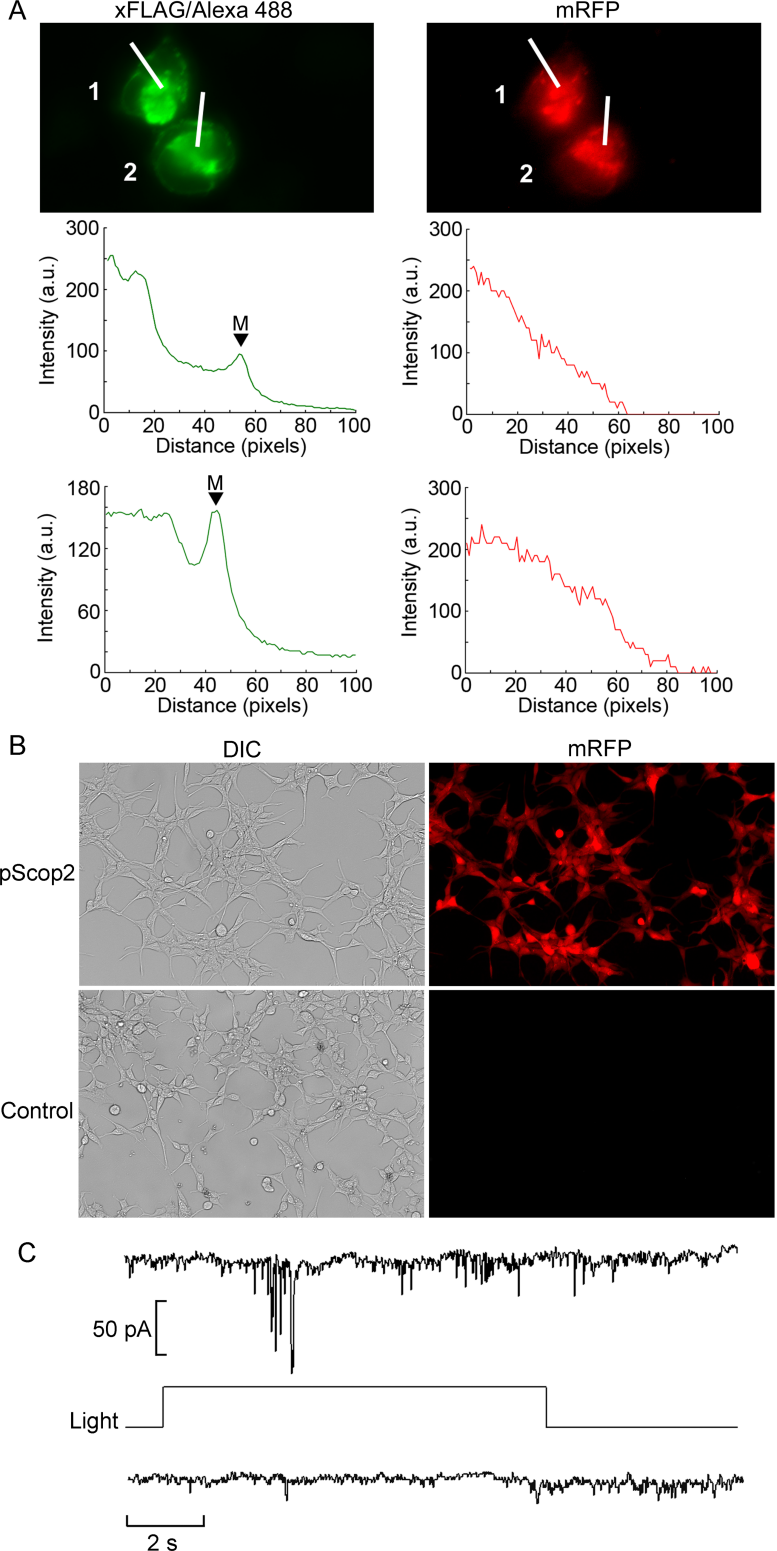
Functional expression of pScop2 in a HEK 293 cell line stably transfected with a bi-cistronic vector. (A) Immunofluorescence of FLAG (left) and mRFP fluorescence (right) showing co-expression of the opsin and the reporter. Intensity profiles were obtained along the lines crossing the two cells, showing a different sub-cellular distribution in the two cases: pScop2 was abundant in the nuclear region, and also accumulated in the membrane (‘M’ & arrowheads), whereas mRFP was more distributed. (B) Low-magnification Nomarski and fluorescence micrographs of the reporter fluorescent protein of a transfected culture subjected to geneticin selection, and control cells. Percentage transfection in the former was 100%. (C) Recording of ion currents in the whole-cell modality, in a stably transfected HEK 293 cell pre-incubated with 11-*cis*-retinal to regenerate the photopigment. Presentation of a light step evoked a fluctuating inward current (top trace). In the absence of lightcurrent remained stable (bottom).

After incubation with the chromophore, transfected cells identified by fluorescence were targeted for electrophysiological recording. Upon attaining the whole-cell configuration, a red light (λ>620 nm) was applied for 5 sec, to further promote regeneration of the R-state of the pigment; this was followed by 5 minutes of dark-adaptation before testing photoresponsiveness. Membrane current was measured at a holding voltage of -50 mV. Initial attempts with transient expression of the pScop2-mRFP fusion construct failed to reveal light-activated changes in membrane current. Two plausible factors may have hindered photoresponsiveness: in the first place, the prior irradiation with bright epifluorescence light may have left the cells desensitized, in spite of the precautions of interposing a photoregeneration and a dark-adaptation period. Second, the opsin fused to the reporter may be intrinsically poorly functional due to steric hindrance problems. To overcome potential shortcomings of the fusion construct we made a bi-cistronic vector (see Methods), and examined separately the expression of mRFP–directly, by its fluorescence–as well as that of pScop2 using anti-Flag antibodies; complete concordance was seen across cells, as illustrated in (Fig 3A), indicating that in successfully transfected cells both transgenes express in a comparable way. Predictably, though, the spatial distribution differed. Whereas, as a soluble protein, mRFP fluorescence was rather diffuse, in the case of pScop2 a more localized pattern was observed: expression was prominent in the nuclear region, with additional accumulation at the plasma membrane, as corroborated by the line profiles displayed in (Fig 3A). With this vector, the efficiency of transient expression in HEK 293 cells was comparable to that obtained with the pcDNA3-mRFP plasmid incorporating the fusion construct. Finally, to avoid the potentially harmful effects of the epi-illumination in the fluorescence selection of target cells, we resorted to a cell line stably transfected with the bi-cistronic vector. (Fig 3B) shows DIC and fluorescence micrographs of transfected and control cultures, demonstrating that 100% of the cells in the transfected culture express the mRFP marker, whereas control cultures viewed under identical conditions were devoid of label. As a result, the selection process prior to electrophysiological measurements can be bypassed altogether. We proceeded with the electrical recording assays: cells were incubated with the chromophore, and subjected to whole-cell recording of membrane current at a fixed membrane potential of -50 mV. After a 5 min dark-adaptation period, a light step was presented; under these conditions, light-dependent currents could be obtained, as illustrated in (Fig 3C), albeit in a minority of instances (n = 3 out of a total of 15 cells tested). In all successful cases, the chromophore utilized was 11-*cis*-retinal.

### Swapping G-protein interaction domains

**Fig 4:**
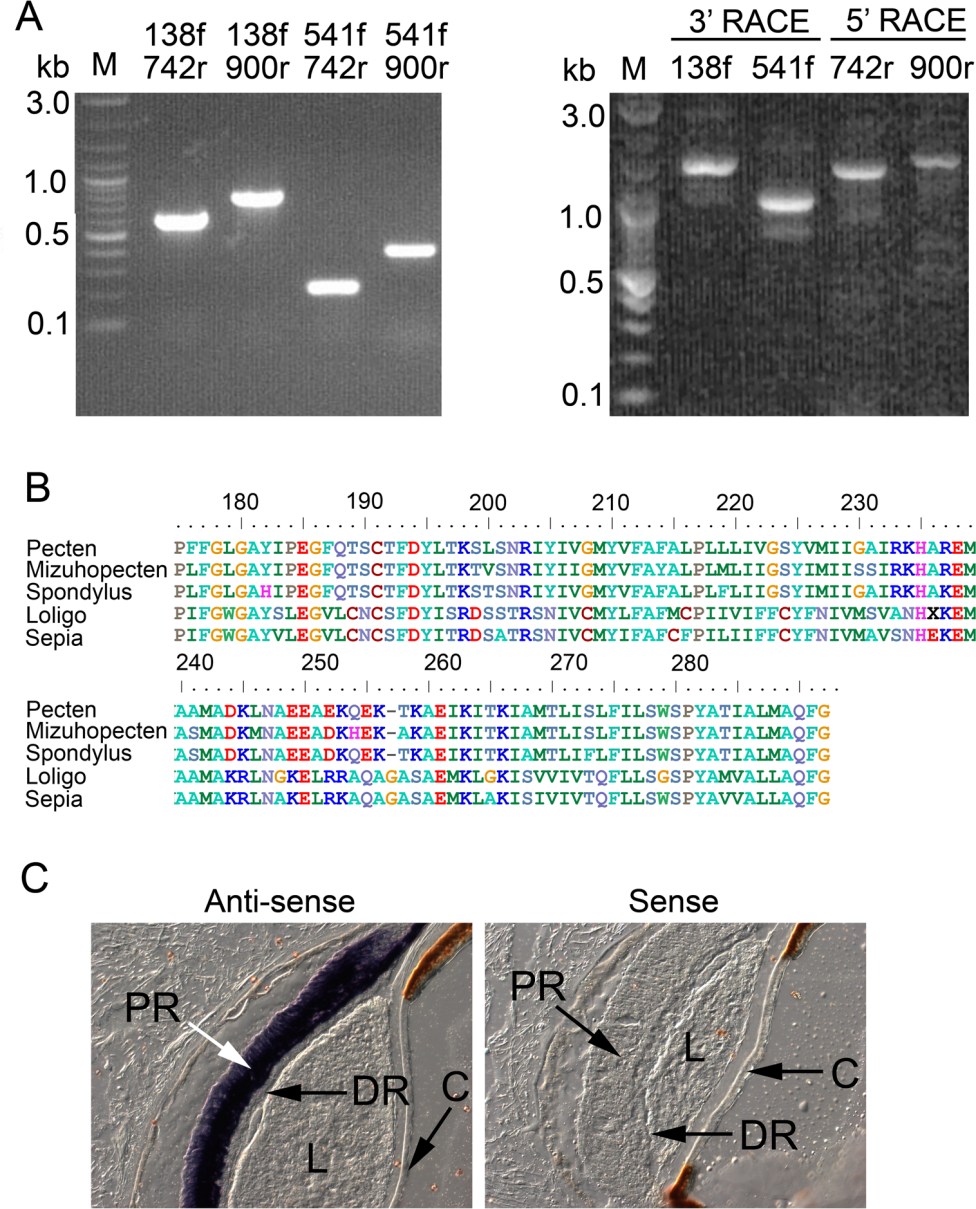
Cloning the G_q_-coupled opsin of *Pecten* microvillar photoreceptors. (A) Products of PCR amplifications obtained with all combinations of four gene-specific primers designed, using the transcriptome, by homology to other R-type opsins (left), and RACE extensions that lead to the full-length clone (right). (B) Alignment of the predicted aminoacid sequence of the core region—which contains critical regions that confer specificity for interaction with G_q_—with the corresponding region of other molluscan R-opsins. (C) *In-situ* hybridization on *Pecten* eye section localizes the transcript of the R-opsin to the proximal layer of the retina, where microvillar photoreceptors are found, while the distal layer of ciliary photoreceptors is completely spared. (L = lens; C = cornea; PR = proximal retina; DR = distal retina).

An additional likely culprit for the modest photoresponsiveness of pScop2-transfected cells could be a faulty coupling to the endogenous G-proteins of the host. Natively, this photopigment couples to G_o_ [25–27], as explained in the Introduction. In HEK cells mRNA levels of G_αo_, detected by microarrays, are marginal at best [35], and even if an endogenous G_o_ was available, sequence divergence with respect to *Pecten* G_o_ (identity ≈82%; [27]) could compromise the productive interaction with pScop2; moreover, no electrophysiological responses attributable to G_o_ activation are known in this cell line. Alternatively, it could be that activated pScop2 taps into a different heterotrimeric G protein of the host cell, but, again, this might entail poor coupling efficiency, and/or physiological effects that only marginally alter membrane conductance. So, a reliable receptor-G protein interaction leading to a defined physiological response is required. G_q_ is abundantly expressed in HEK cells, along with PLC [35], and robust calcium mobilization is readily obtained by activating this phosphoinositide pathway [36, 37]. Therefore, we generated a modified form of pScop2, in which the third intracellular loop (ICL3) was replaced, in order to enhance its competence to tap into G_q_. The G_q_-coupled receptor that served as donor was the opsin of the microvillar photoreceptors that comprise the proximal layer of the double retina of *Pecten*, and which are known to respond to light mobilizing Ca via the G_q_/PLCβ pathway [38]. We cloned such opsin by PCR: having amplified a product homologous to the putative R-opsin previously identified in the Japanese scallop [26], we extended it by RACE (Fig 4A), obtaining overlapping amplicons that, upon CAP assembly, spanned 2168 bp, comprising a full-length transcript (Genebank accession number MG674156). The sequence contains an ORF of 1368 bp, predicting a polypeptide of 456 AA, highly divergent from pScop2 (only 21% identity at the aminoacid level, after CustalW alignment), but very similar to other 'classical', G_q_/PLC-linked invertebrate photopigments: predictably, those of other bivalves from the *Pectinidae* family (*e*.*g*. *Mizuhopecten yessoensis*86% identity) but also the rhodopsins of several cephalopods (50–55% identity); the similarity is especially pronounced in the core region, as illustrated by the alignment in (Fig 4B).To localize its expression, ISH assays were conducted on transversal cryo-sections of fixed retinae of *Pecten*. (Fig 4C) shows a striking labeling pattern, wholly circumscribed to the proximal retinal layer- where microvillar photoreceptors are located—and distinctively sparing the distal layer of the ciliary photoreceptors; this observation attests the complete segregation of the two photopigments in the two classes of visual cells. This opsin will be henceforth referred to as pScop1, following the nomenclature introduced by [26] for the related species, *Mizuhopecten yessoensis*.

**Fig 5:**
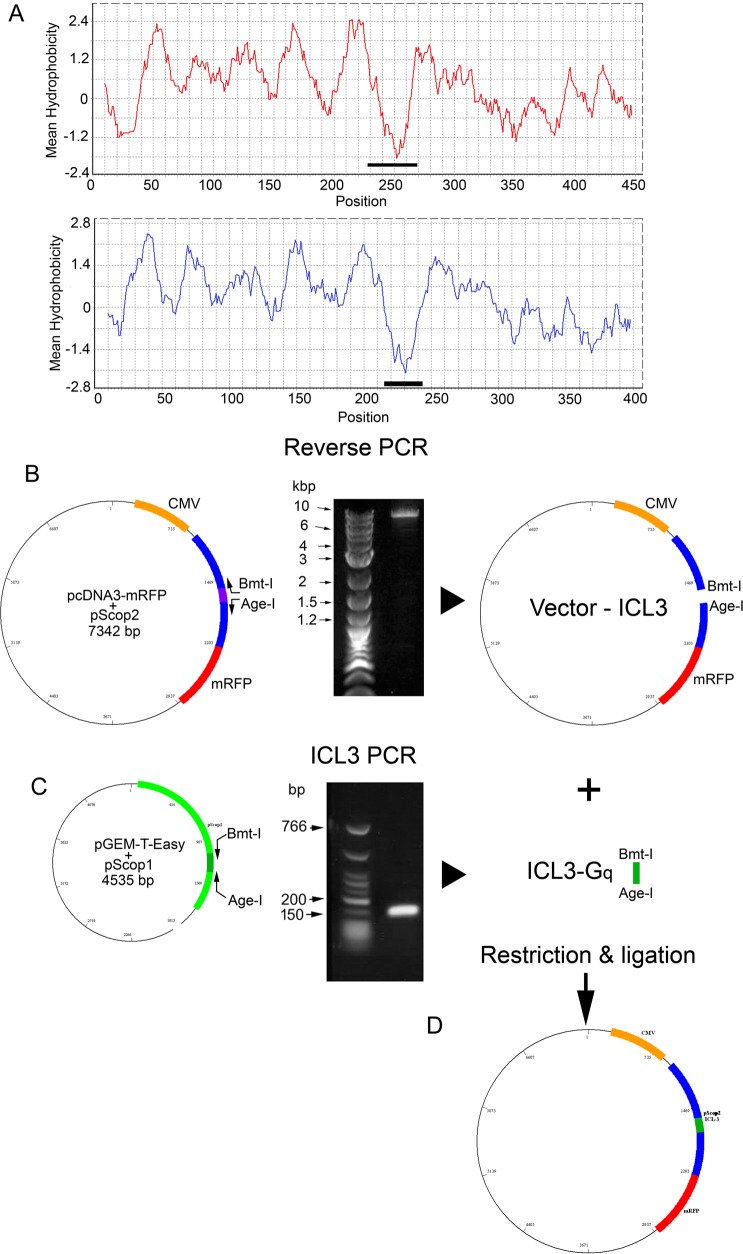
Construction of an expression plasmid containing a modified version of pScop2. (A) Hydropathicity profiles of the predicted aminoacid sequences of the G_q_-coupled opsin of microvillar photoreceptors (pScop1, top), and that of the G_o_-coupled opsin of ciliary photoreceptors (pSco2; bottom). The horizontal black bars mark their respective third intracellular loops (ICL3). (B) Elimination of ICL3 from the bi-cystronic vector encoding pScop2 and mRFP. This was accomplished by a reverse PCR in which the outward-facing primers (arrows) also introduced two unique restriction sites (*Bmt*-I and *Age*-I) to the ends of the amplicon (left). A gel demonstrating a band of the expected size (middle), and a representation of the ICL3-less product (right) are also shown. (C) Amplification by conventional PCR of the G_q_-specific ICL3 using as template another plasmid that contained the sequence of pScop1. The same unique restriction sites were incorporated by the primers. (D) Ligation of the two products, generating a plasmid encoding the chimeric construct.

We then proceeded to replace ICL3 of pScop2 with that of pScop1.The boundaries of ICL3 in the two opsins were estimated from hydropathicity profiles via the Kyte-Doolittle algorithm [39] using an 18-residue window (Fig 5A); the selected stretches (indicated by the horizontal bars) correspond to positions 643–725 in the ORF of pScop2, and 682–804 for pScop1. Deletion of ICL3 from the bicistronic vector containing pScop2 and mRFP was accomplished by a reverse PCR, which simultaneously introduced two unique restriction sites (Fig 5B; see Methods for details); the ICL3 of pScop1 was amplified from another plasmid, adding the same restriction sites (Fig 5C). The two products were ligated, and the resulting chimeric vector (Fig 5D) was dubbed pcDNA3-mRFP-pScop2-G_q_, or pScop2-G_q_, for short.

### Heterologous expression of pScop2-G_q_

**Fig 6:**
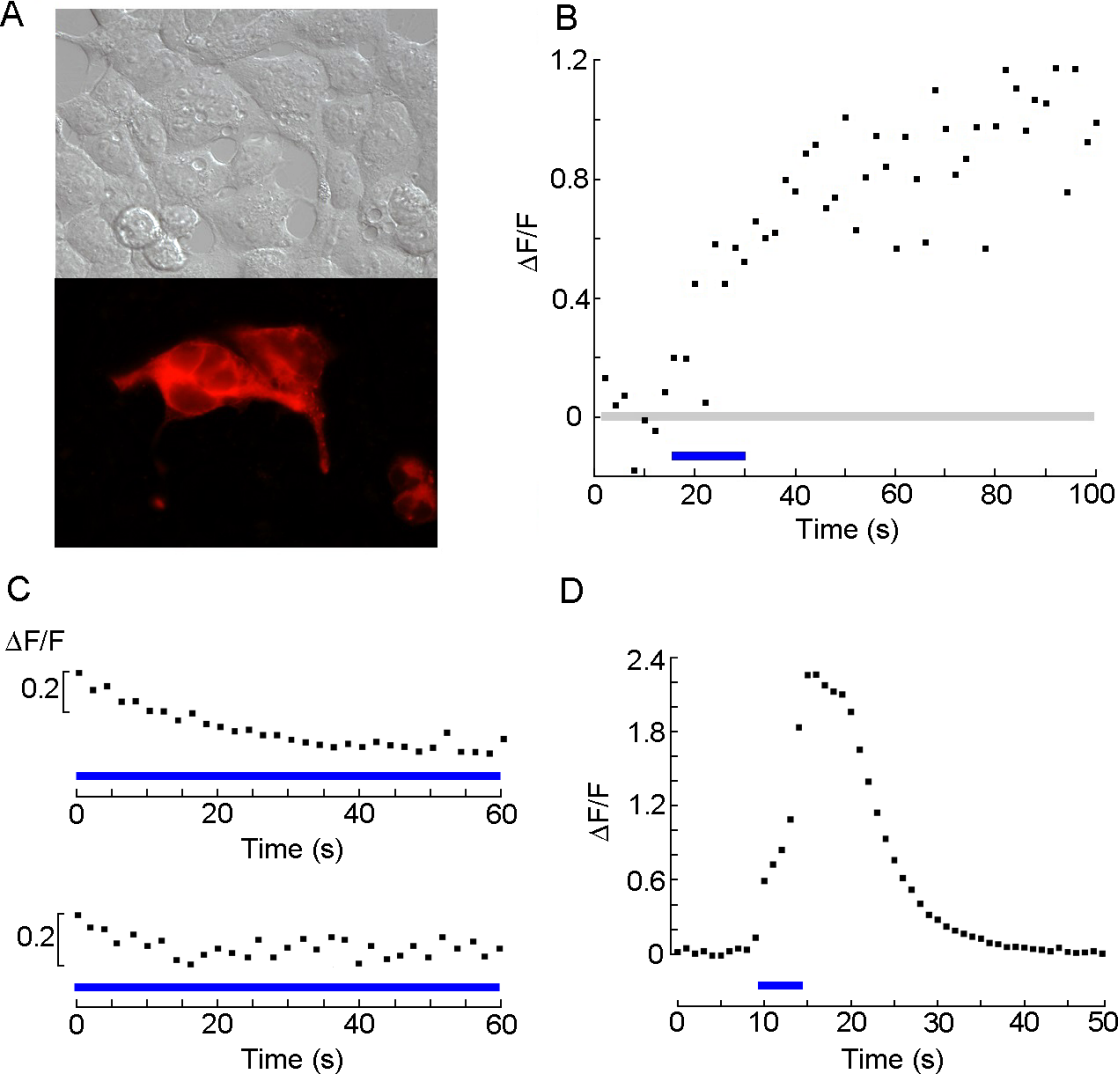
Functional expression of chimeric construct of pScop2 containing a G_q_-specific ICL3. (A) DIC and fluorescence microscopy of HEK-293 cells transfected with the bi-cistronic vector, showing a diffuse distribution of the mRFP. (B) Calcium fluorescence measurements in Fluo 4-loaded transfected cells. Brief pulses of epi-fluorescence illumination were delivered at 0.5 Hz, and fluorescence was repetitively monitored by a photomultiplier during those episodes; after 15 seconds, the same a light was applied in a sustained fashion (blue line) and subsequently the intermittent illumination protocol was resumed. The prolonged light induced a conspicuous increase in Ca fluorescence. (C) Control measurements in which Fluo-4 florescence was monitored in wild-type HEK293 cells incubated with 11-*cis*-retinal (top), or in HEK cells expressing pScop2-G_q_ but not exposed to the chromophore (bottom). A more prolonged irradiation with light, spanning the whole recording interval, was applied, and yet there was no indication of fluorescence increase.(D) Positive control, in which PLC-dependent Ca mobilization was activated by stimulating endogenous G_q_-coupled muscarinic receptors with a saturating concentration of carbachol (20 μM). Fluorescence during the epi-illumination pulses was integrated.

Twenty-four hours after transfection with pScop2-G_q_, HEK cells displayed a pattern of fluorescence indistinguishable from that obtained with the unmodified vector (Fig 6A). For functional assays, we turned to calcium fluorescence, an approach that provides a straightforward read-out of PLC activation, and is less invasive and more expeditious than whole-cell clamp recording. The calcium indicator was Fluo-4, whose peak absorption wavelength is close to that of pScop2, so the same 480 nm light source served the dual function of stimulating both the indicator and the photopigment. In HEK cells Ca-mobilization after PLC activation follows a slow time course spanning tens of seconds [37, 40]; in order to reduce adverse effects of prolonged exposure to the epi-fluorescence light—including bleaching of the calcium indicator—the illumination was discontinuous [41], consisting of repetitive 40 ms flashes delivered every 2 seconds (thus reducing effective irradiation by 98%); fluorescence was sampled concomitantly, during such episodes. Trains of such brief flashes produced little or no change in the level of calcium fluorescence; this is not unexpected, considering the low expression levels of the heterologously expressed photopigment—compared to the dense packing of rhodopsin in the folded membrane of native photoreceptors—so that quantum catch is likely to be very modest. To evoke a response, a more sustained application of the same light (10–15 s), similar to that used in the patch-clamp experiments (see Fig 3C) was utilized to stimulate pScop2, after which the pulsed illumination regime was resumed. A large fraction of the cells transfected and incubated with 11-*cis* retinal displayed a marked increase in Ca fluorescence after the stimulus (57%, n = 19); an example is shown in (Fig 6B). In untransfected controls incubated with the chromophore the fluorescence level failed to raise, even when using a more prolonged light stimulus (Fig 6C, top; n = 6); similarly, cells expressing pScop2-G_q_ but not exposed to retinal failed to show a light response (Fig 6C, bottom; n = 6); in all control cells the trend was downward and typically quite small (probably reflecting the extent of photobleaching of the fluorescent indicator): comparing the fluorescence at the beginning and end of the recording period, the mean ΔF/F (pooled) was -0.17±0.16. Attempts with 9-*cis*-retinal to re-constitute the photopigment were less successful (only 1 cell out of 21 displayed a ΔCa comparable to those of responsive cells treated with 11-*cis*-retinal, 3 more produced marginal responses). No photoresponses were obtained in cells incubated with *all-trans* retinal (n = 10). The light-induced mobilization of internal calcium was compared to that obtained by pharmacological stimulation with saturating doses of carbachol (20 μM); the latter was approximately twofold larger (mean ΔF/F = 1.54 SD = 0.58, n = 6).

## Discussion

The findings of the present study add a decisive piece of information to the growing evidence that this novel group of opsins indeed represents a third lineage of functional visual pigments. Moreover, they buttress the viability of utilizing rhodopsins as potential optogenetic tools to exert control by light over G-protein signaling cascades—much like channelopsins have been employed to photo-manipulate membrane voltage. Two important features of an implantable photopigment are that it should not bleach with light (unlike vertebrate rhodopsins) and should be readily amenable to heterologous expression (unlike most opsins of invertebrates). We were able to express pScop2, together with fluorescent reporter, in all the mammalian cells lines tested, both as a fusion construct, as well as independently via a bi-cistronic vector. This indicates that the hurdles normally encountered with R-opsins are not as severe in the case of G_o_-coupled opsins, perhaps owing to their relative closeness to their vertebrate counterparts [16]. Nonetheless, the efficiency of lipofectamine-mediated transfection was generally modest (up to ≈20%, as assayed by fluorescence microscopy); future efforts will strive to optimize conditions for attaining a higher effectiveness, but for the purpose of exploring the possibility of conferring photosensitivity to a host cell, such expression levels were adequate. The negative results in initial attempts of functional reconstitution using transient expression of fusion constructs were likely due to (i) the need to select target cells by fluorescence microscopy, where the strong epi-illumination could compromise photopigment integrity, or cause a loss of sensitivity that the subsequent period of dark adaptation could not restore. (ii) Interference of the fluorescent protein with the functionality of the photopigment. To address both potential shortcomings, a cell line stably transfected with the bi-cistronic vector was generated; under those conditions, success rate increased and several examples of photocurrent of small amplitude were documented; the improvement, though, remained rather marginal. This prompted the question of whether the coupling of pScop2 to endogenous effectors may be at fault. pScop2 and cognate opsins of other *Pectinidae* natively couple to G_o_, as indicated by (i) the co-localization with G_o_, and absence of other G-proteins in the distal retina layer that contains ciliary photoreceptors [26,27]; (ii) the effectiveness of pharmacological manipulations designed to target G_o_ [25]; (iii) the selective inhibition of the photoresponse by siRNAs-mediated knock-down of G_o_ [27]. G_o_ is the most abundant G-protein in the vertebrate brain [42], but it is poorly expressed in HEK cells [35]; furthermore, its diverse effectors for the most part remain unknown. While our exploratory measurements had demonstrated robust ion currents evoked by GTP-γ-S (see Fig 2), this agent is a non-selective G-protein activator; as such, there was no guarantee that pScop2 would efficiently couple to the same endogenous pathways that control such conductances: although cross-activation between a GPCR and different G-proteins subtypes has been reported [43–45], such promiscuity is limited, and little or no crosstalk occurs for some receptor/G-protein combinations [46]. We therefore opted for re-directing pScop2 signaling towards another G-protein pathway with a well-defined physiological response, a strategy that proved successful with other opsins [47]. In HEK-293 cells a G_q_ is recruited by stimulation of muscarinic receptors of the M1 and M3 sub-types, as well as bradykinin and P1/P2 purinergic receptors [36, 37, 40]; activation of such receptors leads to PLC-dependent mobilization of internal Ca. To improve the likelihood that pScop2 may engage the G_q_ of HEK cells, we substituted its third intracellular loop; this region is important for determining specificity of interaction of 7TMDRs with G-protein subtypes [48–50]. The source of the transplanted G_q_-specific ICL3 was the cloned opsin of another photoreceptor type present in the same retina; this belongs to the class of R-opsins, and signals through PLC. By monitoring light-induced Ca mobilization by fluorescence, we found that photosensitivity in the cells transfected with the chimeric construct improved dramatically, with nearly 60% of them exhibiting a clear response upon illumination. This represents the first successful functional expression of an opsin of this class in a heterologous system. The smaller size of the response to light, compared to the activation of the G_q_/PLC cascade via endogenous muscarinic receptors, may be due to different factors. On the one hand, it could reflect differences in expression levels of the implanted *vs*. the native receptors; on the other hand, it is worth stressing that while ICL3 is an important determinant for G-proteins recognition, it is not the only region involved, and portions of ICL2 and ICL4 also participate (reviewed by [28]). One can therefore envision a more thorough re-engineering of pScop2 to achieve optimal coupling to different G proteins and their associated signaling pathways, paving the way for a host of variants with a great potential for expanding the range of control of cellular functions. On the other hand, the possibility of tuning the spectral sensitivity by point mutations [51] may also help tailor the characteristics of the implanted photopigment to particular applications and optimize its effectiveness.

Most successful trials utilized 11-*cis*-retinal as chromophore. At present, the reasons for such differences are not clear: the β-ionone ring of 9-*cis*-retinal, critical for the formation of the Schiff base linkage to the opsin moiety, is identical to that of 11-*cis*-retinal, and, in spite of the shorter polyenic chain, its photoisomerization can dutifully reproduce the native light response—at least in some systems [33, 52–54]. On the other hand, there was some expectation that externally supplied *all-trans* retinal might have worked to reconstitute this photopigment, owing to its thermal stability; in fact, such possibility has been verified *in vitro* for Amph OP1 [55], but that did not seem to be the case for pScop2. The apparent failure of these two retinals remains to be investigated.

The fact that pScop2 is thermally stable—a feature shared by all invertebrate photopigments—has some appealing consequences for optogenetics, where photopigment re-generation can constitute a key bottleneck. It is true that certain non-photoreceptor host cells express the metabolic machinery for retinoid processing (including certain HEK 293 strains; [56, 57]), and in some instances light responses arising from an implanted photopigment have been obtained relying solely on available retinal, without any exogenous administration (*e*.*g*. [58]. Nonetheless, if the chromophore has to be replaced after each episode of illumination, supply can be rate-limiting, resulting in slow and/or incomplete regeneration and degraded responsiveness to repetitive stimuli. Photopigments that do not bleach upon photo-activation ease worries about rapid recovery, and may only require 'maintenance-level' supply of chromophore. As for the bi-stability—an attribute that is only found in a subset of non-bleaching photopigments—the possibility of using chromatic illumination to impose a given distribution of photopigment states provides obvious benefits, allowing the activation and de-activation of the target G-protein cascade. In panopsins the shift in peak absorption wavelength caused by photoisomerization is quite small (≈10 nm; [22]); as a result, photopigment states can be manipulated only to a marginal extent. By contrast, massive ‘on’/’off’ re-arrangements have long been known to occur in a number of invertebrate photoreceptors, like the UV photoreceptors of the frontal ocelluls of *Limulus* [59], the giant photoreceptors of the primitive eye of *Balanus* [60], and the photoreceptors of *Drosophila* [61]. However, the amenability of pScop2 for expression in mammalian cells confers a special appeal to this opsin—and, most likely, to other G_o_-linked opsins. In fact, this may inspire similar efforts to investigate ciliary photoreceptors of other bivalves such as *Lima scabra* [62], that also exhibit a prominent photoresponse bi-stability, and whose photopigments—as yet unidentified—may offer other advantages. We did not attempt to corroborate the bi-stability of the heterologously expressed pScop2; to do so by means of electrophysiological tests requires fulfilling special conditions which exist in native photoreceptors, but may be hard to realize in expression systems. One approach consists in recording early receptor potentials or currents–which report the minute charge displacements associated with photopigment conformational changes [63]–and demonstrating that their polarity can be inverted with suitable manipulations of the wavelength of adapting and test lights [34]. Another strategy is to use intense stimuli of different chromatic content to evoke and terminate prolonged aftercurrents [59, 60, 62]. These occur when the amount of activated photopigment overwhelms the mechanisms that inactivate it (*e*.*g*. arrestin; [64]); as a result, stimulatory effects can linger—from minutes to hours—until another chromatic light stimulus re-sets the photopigment to the inactive state. In both the above cases, a massive expression of the photopigment is required (like in true photoreceptors, where their infolded membrane can accommodate large amounts of rhodopsin); the occurrence of prolonged aftercurrents, in addition, hinges on the interplay with de-activation mechanisms of the signaling pathway, otherwise this particular consequence of bi-stability may not become manifest. Nonetheless, the ability to turn on- and off- the initial link of the cascade remains an attractive feature that can add versatility to this optical approach for controlling cellular functions. While vertebrate visual pigments do not offer such versatility, interesting progress has recently been reported with mammalian melanopsin, the G_q_-linked photopigment of 'circadian' light sensors: blue-light irradiation of the heterologoulsy expressed receptor was shown to activate also G_i/o_; remarkably, slow de-activation could be the obtained with longer wavelength illumination [65].

Finally, it must be pointed out that efforts to control chemical cascades by light have not been circumscribed to the use of G protein-coupled photopigments: for example, the photo-activated adenylate cyclase from the flagellate *Euglena gracilis* has been utilized a to alter cAMP levels in transfected cells, and even cause light-dependent behavioral changes in *Drosophila* expressing the transgene in neurons [66]. Similar manipulations have also become possible with cGMP [67], either by altering the catalytic site of photosensitive ACs via site-directed mutagenesis, or using recently uncovered natural photo-activated GCs, such as that isolated from the fungus *Blastocladiella emersonii*. Moreover, an array of promising optogenetic tools are being engineered using diverse non-retinal based photoreceptor molecules as templates [68].

## Materials and methods

### Cloning and domain-swapping

RACE-ready cDNA from *Pecten* retinae was prepared using the Clontech SMART RACE kit; PCR amplifications and RACE extensions were conducted as previously described [64]. For Sanger sequencing, products were ligated into pGEM-T-Easy (Promega) used to transform JM109 bacteria (Promega), which were then cultured in X-Gal/IPTG agar dishes, following standard procedures. Plasmids were extracted and purified with the Qiaprep kit (Qiagen). To replace a selected stretch of the photopigment sequence with that of another, a plasmid containing the donor sequence served as template; primers incorporating suitable unique restriction sites in their 5' end (*Bmt-I* and *Age-I*) were used in a touch-up PCR protocol (necessary because of the presence of 12 non-complementary bases in the primers), and the resulting product was ligated into a pGemT-easy vector and amplified in transformed bacteria, followed by plasmid extraction and purification; the identity of the insert was corroborated at various stages by colony PCR, restriction analysis, and sequencing. In parallel, an inverse touch-up PCR [69] using the acceptor plasmid as template eliminated the undesired portion, while adding the same two restriction sites; the amplicon was gel-purified and phenol-chloroform-extracted. Donor and acceptor were then doubly-restricted with the appropriate endonucleases (New England Bio Labs), and the desired digests were gel-purified, mixed, ligated (T4 Ligase, Promega, 12 hrs at 4°C), and used to transform J109 bacteria. Confirmation of the deletion of the native stretch and its replacement with that of the donor was based on restriction analysis with enzymes expected to work only if the swap had successfully taken place, as well as on PRC amplification and sequencing of relevant portions of the plasmid.

### *In-situ* hybridization

The template for generating the ribo-probes was a dual-promoter expression vector (pBluescript IISK (+), Stratagene) into which the target sequence had been directionally inserted, via restriction with Sac I and Sac II and ligation. After linearizing the plasmid with either one of the two enzymes, sense and anti-sense RNA probes were generated, using T3 or T7 RNA polymerase (2 hrs at 42°C) and digoxygenin-labeling mix (Roche). The probe was EtOH-precipitated, dried, re-suspended in DEPC H_2_O and its concentration was determined spectro-photometrically prior to dilution in hybridization buffer as a 10× stock, to be stored at -20°C. A dot-blot was carried out in a nylon membrane, to determine labeling efficiency. The hybridization protocol was described previously [64]. The probes were detected with anti-DIG antibodies (Roche) conjugated to alkaline phosphatase (AP; 1:2000), and visualized by development with AP substrate solution (BM Purple, Roche).

### Heterologous expression

The pScop2 sequence [27] was modified in the following ways: (i) A 3’ terminal sequence encoding a Flag epitope was added. (ii) The coding portion was codon-optimized for expression into human cells, with the re-synthesized construct inserted into the pUC57-Kan vector (Genewiz). For transfecting cell lines, two expression vectors, V7 XLT.GFPLT CS2+ and pcDNA3-mRFP (Addgene, Cambridge, MA) were utilized. These encode a variant of the green fluorescent protein, GFP, and the monomeric form of the red fluorescent protein, mRFP, respectively, located downstream of the multicloning site, so to allow the generation of a fusion protein with the reporter in the carboxy region. In order to transfer the codon-optimized version of pScop2 into the new vectors, restriction sites were added to it for in-frame directional ligation, by re-amplifying the insert from pUC57-Kan with primers encoding the appropriate sequences in their 5’ end. The forward primers were designed to target the pScop2 5’-UTR 90 bases before the ORF, and the reverse primers to delete the Scop2 stop codon. For the vector V7 XLT.GFPLT CS2+ the restriction sites introduced were *Bam*HI and *Cla*I, whereas for pcDNA3-mRFP *Hind*III and *Xho*I were used instead. Both vectors and PCR products were subjected to a double digestion for 2–3 hrs at 37°C, terminated by incubation at 65°C for 20 min to inactivate the enzymes. Subsequently, they were gel-purified and quantified spectrophotometrically before proceeding with directional ligation. The resulting two constructs were verified by sequencing to corroborate the correct in-frame position of the pScop2 insert. Subsequently, they were amplified by transforming JM109 bacteria: 25 ml of selective LB medium were inoculated and grown for 16 hrs a 37°C with strong agitation until O.D._600_ reached 1.8–2.4. Plasmid DNA was extracted and purified (Quiagen Midi) for storage at -20°C in TE buffer.

In case fusion constructs turned out to be non-functional because of interference or steric hindrance by the reporter, a bi-cistronic vector was also constructed, to express pScop2 separately from the fluorescent protein. To this end, an IRES (**I**nternal **R**ibosomal **E**ntry **S**equence) was added at the 5’ end of Scop2, and a second one at the 3’ end, after the stop codon. This sequence permits the assembly of the ribosome machinery independently of the interaction of the mRNA with CAP binding proteins and eIFs (**e**ukaryotic **I**nitiation **F**actors), thus facilitating translation of the mRNA. So, with the mRFP coding sequence located downstream of pScop2, the second IRES will cause it to express. The ORF of pScop2 remained essentially unchanged, except for the aforementioned FLAG epitopes and a poly-His epitopes, separated by glycines, before the stop codon. *Hind*III, *Bam*HI and *Eco*RI recognition sequences were also added at the 5’ end of the chimera, while *Eco*RI and *Sac*II were introduced immediately before the second IRES sequence; finally, *Eco*RV and XhoI were incorporated at the 3’ end. This construct was ligated into pcDNA3-mRFP after restriction with *Bam*HI and *Eco*RV.

The cell lines tested were: HEK293 (a generous gift of Dr. Walter Stühmer, Max Planck Institute for Experimental Medicine, Göttingen), N2A (ATCC CCL-131), CHO (European Collection of Cell Cultures CB2475) and LLC-PK1-CL4 (generously provided by Dr. James Bartles, Northwestern University). Cells were cultured at 37°C in 5% CO_2_, in media supplemented with 10% FCS; the media were high-glucose DMEM for HEK293 and N2A cells, nucleoside-free αMEM for LLC-PK1-CL4, and F12 for CHO cells (all from Gibco/Invitrogen). Initially, media included penicillin (100 u/ml), and streptomycin (100 μg/ml), but in later cultures (including those employed in all functional assays) these were omitted. Cells were transfected with lipofectamine 2000 (Invitrogen) for 4 hrs at 37°C and examined 24–72 hrs later; transfection efficiency in the different conditions was estimated by manually counting cells from multiple micrograph pairs (DIC/fluorescence).

### Generation of stably transfected cell lines

Individual clones were selected by Geneticin (G418, Sigma), since all the expression vectors employed in the present work include the coding sequence for the gene Neo^R^/Kan^R^, which confers resistance to this antibiotic. To determine the appropriate dose for selection, wild-type cells were cultured at different Geneticin concentrations (100–800 μg/ml) and their growth was monitored. At G418 dose > 200 μg/ml HEK293 were unable to grow; a dose of 800 μg/ml was chosen for subsequent selection protocols. Twenty-four hours after transfection, cells were trypsinized and seeded in culture dishes at low density (~1% of confluence) in culture medium supplemented with G148. The medium was changed every 3 days and the formation of fluorescent colonies derived from an individual cell was monitored. Rapidly growing colonies were isolated using a plastic cylinder which was sealed to the bottom of the culture dish by means of sterilized high-vacuum grease (Corning), and trypsinized. Each selected colony was cultured for 4 weeks in the selective medium, after which the antibiotic concentration was reduced to a maintenance level of 600 μg/ml.

### Electrophysiology

For patch-clamp recording, cells were seeded onto pre-cut pieces of #1 coverslip glass, which were transferred to the perfusion chamber mounted onto the stage of an inverted microscope (Nikon) and continuously superfused with Ringer (140 mM NaCl, 1.5 mM KCl, 2.5 mM CaCl_2_, 11 mM Glucose, 10 mM HEPES, 1 mM MgCl_2_, pH 7.4) Patch pipettes were fabricated from thin-wall borosilicate glass and fire-polished before use. The base composition of the electrode-filling solution was (in mM): 110 K-glutamate, 15 KCl, 20 NaCl, 0.5 CaCl_2_, 4 MgATP, 1.5 Na_2_EGTA, 5 mM HEPES and 200 μM GTP, pH 7.3. Electrode resistance measured in Ringer was 2–4 MΩ; series resistance was compensated electronically. Either an Optopatch (Cairn Research) or a custom-built amplifier was used to measure membrane currents. Data were digitized with an analog-digital interface (DT9834, Data Translation, Marlboro, MA), which served also to generate stimuli under the control of software developed in-house.

### Calcium fluorescence

Cells were incubated with 4.5 μM of the fluorescent Ca indicator Fluo-4AM, (Molecular Probes) dissolved in Ringer supplemented with 0.1% Pluronic F-127 and 0.5% DMSO, for 1 hour at 37°C in the dark. Epi-illumination provided by Xenon arc lamp (PTI), an electromechanical shutter (Vincent Associates, Rochester), and an interference filter (Fc ≈ 480 nm, Chroma) was delivered via a liquid light-guide (Oriel) to the epi-fluoresce port of the inverted microscope; a 500 nm dichroic reflector was installed beneath the Nikon 100× 1.4 N.A. oil-immersion objective. An adjustable mask placed at a conjugated image plane restricted the collection of light to a small, defined area to enhance S/N. Output light was split by an additional dichroic mirror (λ_c_ 570 nm): shorter wavelenghts were diverted towards a barrier filter (λ>520 nm) and a photon-counting photomultiplier (Hammamatsu) connected to a pre-amplifier/discriminator and a rate meter (Modern Instrumentation Technology); long-wavelength light was instead directed to an IR-sensitive CCD camera (Genwak), which served to observe and position the target cells as well as the adjustable mask.

### Photopigment reconstitution

The vitamin A-derived chromophores were *all-trans*-retinal (Sigma), 9-*cis*-retinal (Sigma), and 11-*cis*-retinal (generous gift of Dr. Carter Cornwall, Boston University School of Medicine). Before conducting a physiological assay, an aliquot of retinal diluted in EtOH was mixed with Ringer to a final concentration of 10–20 μM, and cells were incubated 1 hour and washed.

*Pharmacological stimulation*. The cholinergic agonist carbachol (Sigma) was dissolved in Ringer solution and applied locally via a 'puffer' micropipette positioned in proximity of the target cells. Pressure ejection was initiated under the control of a solenoid-operated valve.
